# Supplementary material for: Proteomics reveal biomarkers for diagnosis, disease activity and long-term disability outcomes in multiple sclerosis
Source: Nat Commun. 2023 Oct 30;14:6903. doi: 10.1038/s41467-023-42682-9 (PMC10616092; doi:10.1038/s41467-023-42682-9)
Supplement: Supplementary file 1 — Supplementary Information [file 41467_2023_42682_MOESM1_ESM.pdf]

Supplementary Information

Proteomics reveal biomarkers for diagnosis, disease activity and long-term disability outcomes in multiple sclerosis

Julia Åkesson[1,2,†], Sara Hojjati[3,†], Sandra Hellberg[1,3], Johanna Raffetseder[3], Mohsen Khademi[4], Robert Rynkowski[5], Ingrid Kockum[4], Claudio Altafini[6], Zelmina Lubovac-Pilav[2], Johan Mellergård[5], Maria C Jenmalm[3], Fredrik Piehl[4], Tomas Olsson[4,†], Jan Ernerudh[7,†], Mika Gustafsson[1,†,*]

[1] Bioinformatics, Department of Physics, Chemistry and Biology, Linköping University, 581 83 Linköping, Sweden.
[2] Systems Biology Research Centre, School of Bioscience, University of Skövde, 541 28, Skövde, Sweden.
[3] Division of Inflammation and Infection, Department of Biomedical and Clinical Sciences, Linköping University, 581 83 Linköping, Sweden.
[4] Neuroimmunology Unit, Department of Clinical Neuroscience, Center for Molecular Medicine, Karolinska University Hospital, Karolinska Institute, 171 76 Stockholm, Sweden.
[5] Department of Neurology, and Department of Biomedical and Clinical Sciences, Linköping University, 581 83 Linköping, Sweden.
[6] Division of Automatic Control, Department of Electrical Engineering, Linköping University, 581 83 Linköping, Sweden
[7] Department of Clinical Immunology and Transfusion Medicine, and Department of Biomedical and Clinical Sciences, Linköping University, 581 83 Linköping, Sweden.

† These authors contributed equally to the work.
* Corresponding author: mika.gustafsson@liu.se

List of Figures:

Figure S 1. Influence of sampling handling. 3

Figure S 2. A principal component analysis of the plasma samples from persons with MS (pwMS). 4

Figure S 3. Heatmap of differentially expressed proteins in cerebrospinal fluid. 5

Figure S 4. Expression value distribution of the 19 known MS biomarkers. 6

Figure S 5. Heatmap of differentially expressed proteins in cerebrospinal fluid (CSF) and plasma in persons with MS vs. healthy controls. 7

Figure S 6. The correlation between cerebrospinal fluid samples and plasma samples. 8

Figure S 7. Correlations between treatment duration index and protein expression. 9

Figure S 8. Individual plots: expanded disability status scale (EDSS) scores of persons with MS. 10

Figure S 9. Normalized age-related MS score (nARMSS) distribution for persons with MS (pwMS). 15

Figure S 10. Portions of samples below the limit of detection (LOD) in persons with MS (pwMS) and healthy controls (HC). 16

Figure S 11. MS network: circular layout, and KEGG pathway enrichment. 17

Figure S 12. Portion of samples below limit of detection (LOD) in persons with MS (pwMS) and healthy controls (HC): comparison between discovery and replication cohorts. 18

Figure S 13. Singular value decomposition analysis: uncorrected and corrected NPX values in cerebrospinal fluid (CSF) and plasma samples. 19

List of Tables:

Table S 1. Known MS biomarkers with supporting references. 20

Table S 2. Performance of the 52 differentially expressed proteins in cerebrospinal fluid in the discovery cohort for predicting MS diagnosis. 21

Table S 3. Performance of the 52 differentially expressed proteins in cerebrospinal fluid in the discovery cohort for predicting disease activity. 23

Table S 4. A linear regression model consisting of 11 cerebrospinal fluid (CSF) proteins could predict normalized age-related MS score (nARMSS). 25

Table S 5. The performance of the normalized age-related MS score (nARMSS) model after exclusion of three proteins. 26

Table S 6. References for proteins in the MS network that were not annotated with a Gene Ontology term. 27

Table S 7. References for the drug classification into first-line (low-efficacy) and second-line (high-efficacy). 28

References 29

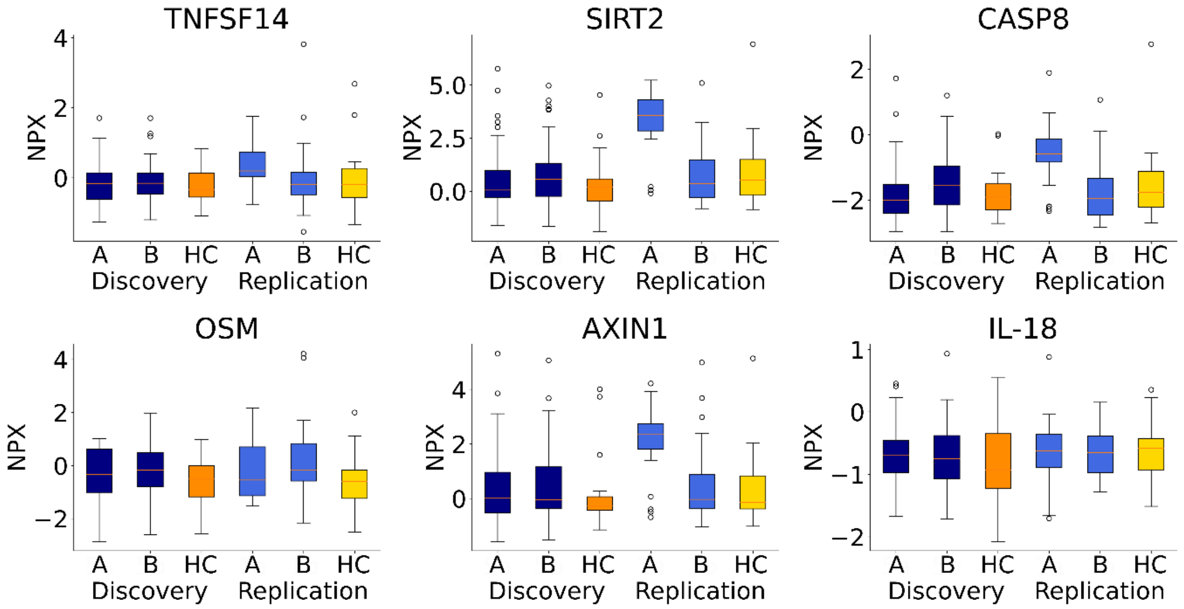

Figure S 1. Influence of sampling handling.

A group (n = 21) of persons with MS (pwMS; replication cohort group A) had higher expression of several protein markers for delayed sampling handling (TNFSF14, SIRT2, CASP8, AXIN1) in plasma. The figure shows the expression of the top six most affected proteins, also measured on the Olink panel, by sampling handling variability from Table 1 in [1]. The pwMS from both the discovery and the replication cohort were divided into two groups (A and B), which in both cohorts represents samples from an old collection (A) and from a new collection (B). The remaining groups of pwMS (discovery cohort group A and B, and replication cohort group B) and the healthy controls (HC) did not show any clear effect of sampling or handling variability.

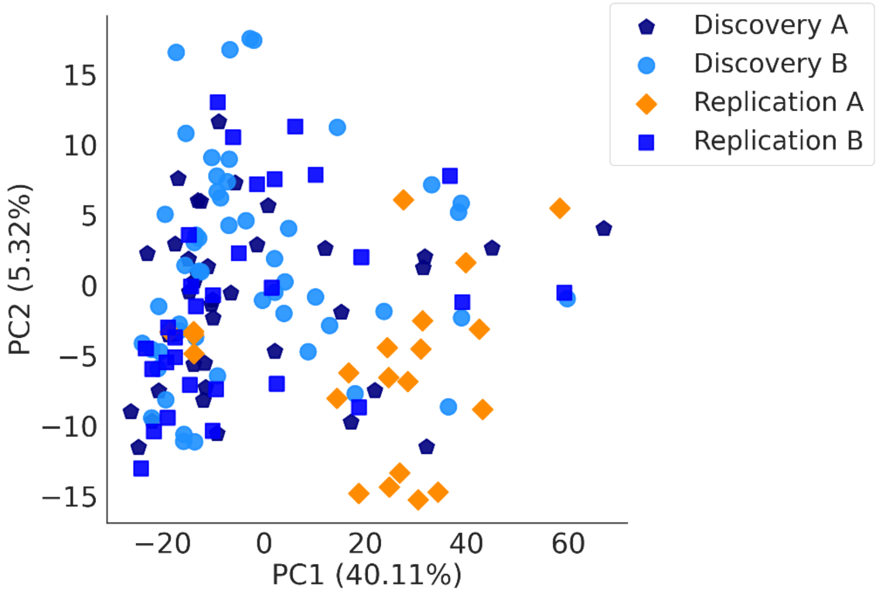

Figure S 2. A principal component analysis of the plasma samples from persons with MS (pwMS).

The plasma samples from one group (replication cohort group A, n = 21 samples) of pwMS are to a high extent clustering separately compared to the samples from the remaining groups of pwMS (discovery cohort group A and B, and replication cohort group B). The pwMS from both the discovery and the replication cohort were divided into two groups (A and B), which in both cohorts represents samples from an old collection (A) and from a new collection (B).

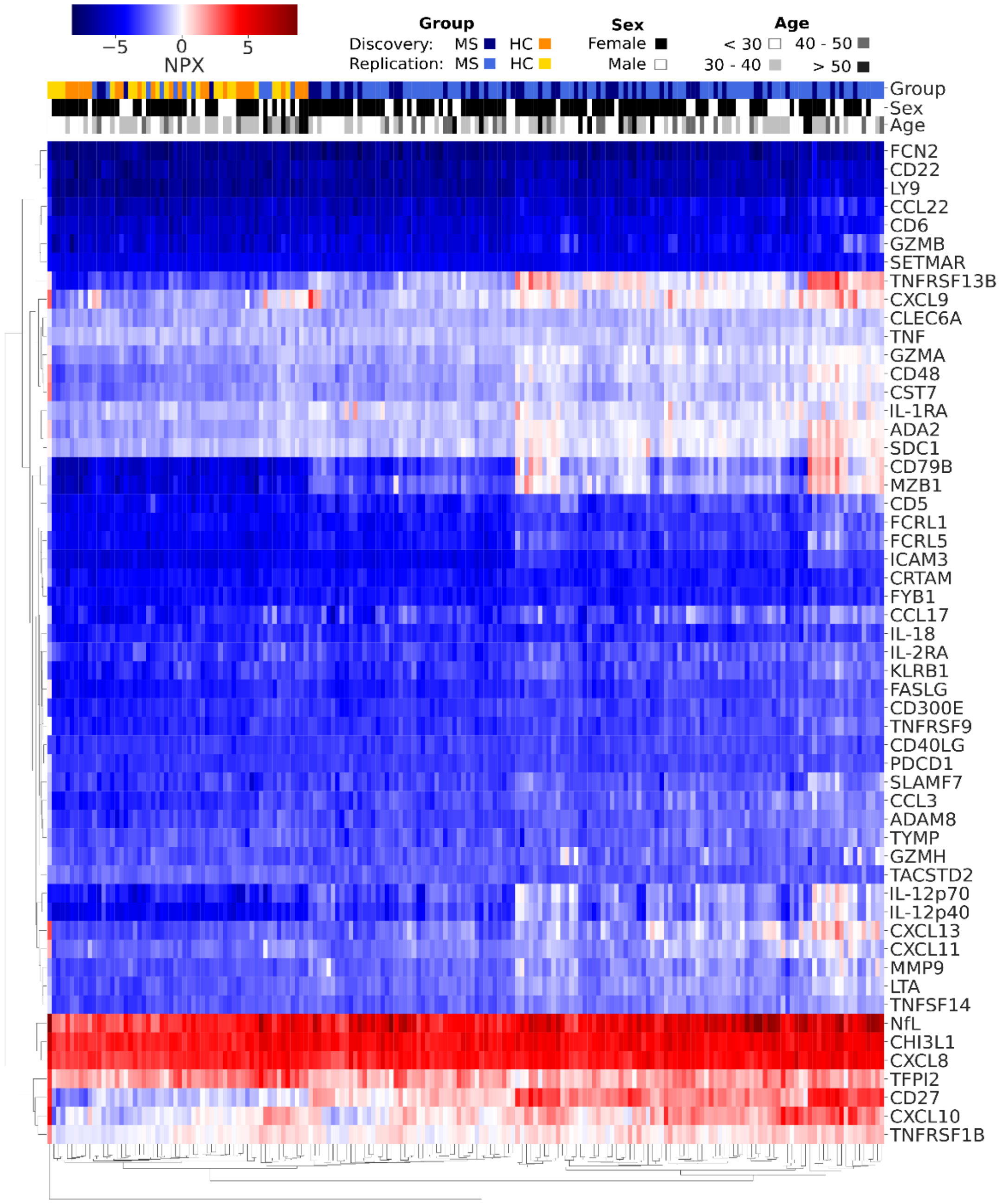

Figure S 3. Heatmap of differentially expressed proteins in cerebrospinal fluid.

A clustered heatmap showing the protein expression (NPX) of the differentially expressed proteins (false discovery rate < 0.05) either in the discovery cohort or the replication cohort in the cerebrospinal fluid. Each column shows the expression of one sample. At the top of the heatmap it is shown which groups a sample is part of (person with MS or healthy control (HC) and discovery cohort or replication cohort), sex (female or male), and age at baseline (16 - 64 years) of each person.

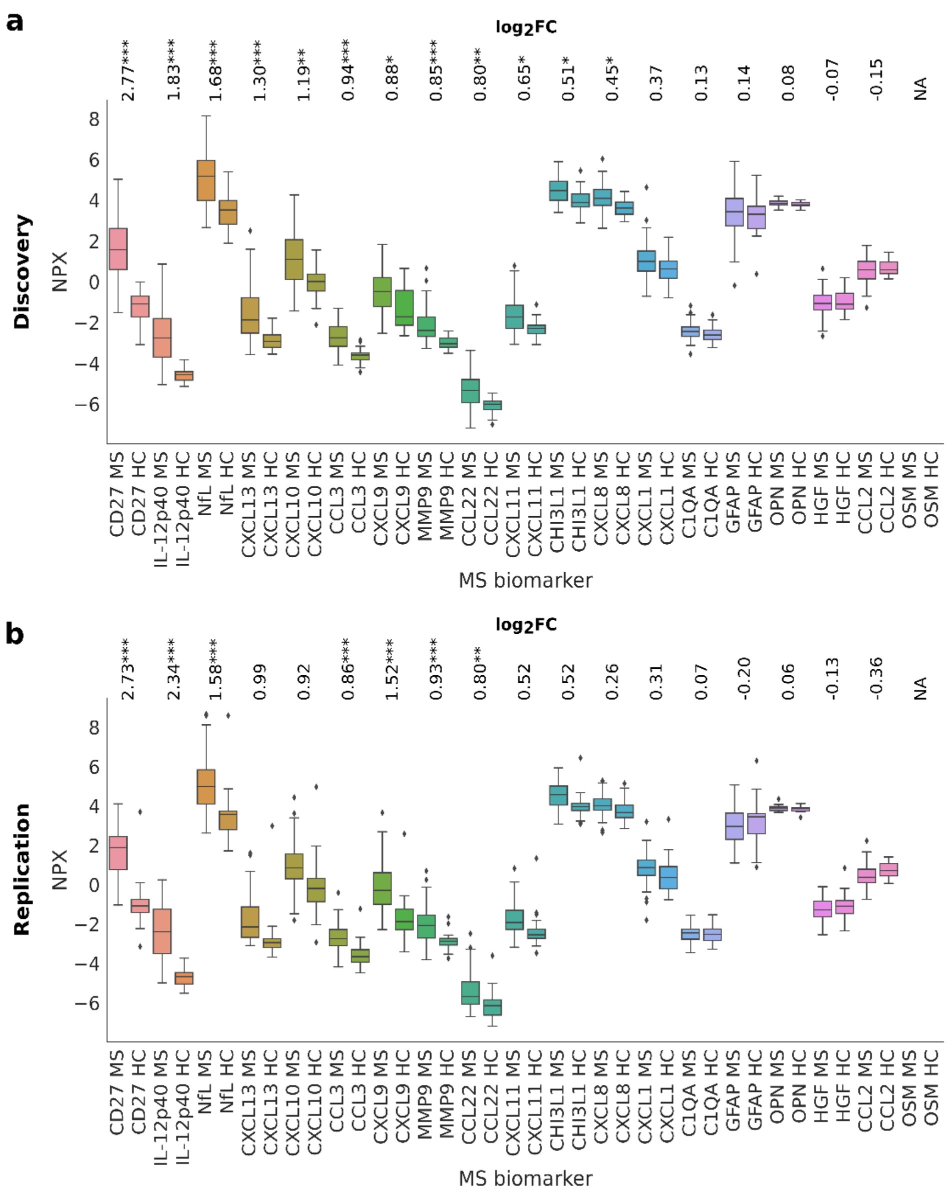

Figure S 4. Expression value distribution of the 19 known MS biomarkers.

The expression value distribution of the 19 known MS biomarkers curated in the present study in **a** the discovery cohort and **b** the replication cohort. The expression value distributions are compared for samples from persons with MS (pwMS) with samples from healthy controls (HC). The log2 fold-change (FC) obtained in the differential expression analysis are stated above the boxes for the corresponding protein. The protein OSM was below the limit of detection in more than 75% of the samples, and were therefore not included in the analysis. * false discovery rate (FDR) < 0.05, ** FDR < 0.01, *** FDR < 0.001. The significance of the $\log_2$FC values was obtained from a two-sided linear model t-test (Limma analysis). The FDR corrected p-values in the order (discovery cohort, replication cohort) were CD27 ($2*10^{-13}$, $2*10^{-10}$), IL-12p40 ($4*10^{-8}$, $5*10^{-10}$), NfL ($2*10^{-5}$, 0.001), CXCL13 (0.001, 0.09), CXCL10 (0.004, 0.17), CCL3 ($1*10^{-6}$, $2*10^{-4}$), CXCL9 (0.02, $4*10^{-5}$), MMP9 ($1*10^{-4}$, $3*10^{-4}$), CCL22 (0.002, 0.07), CXCL11 (0.02, 0.23), CHI3L1 (0.02, 0.07), CXCL8 (0.02, 0.31), CXCL1 (0.40, 0.39), C1QA (0.55, 0.71), GFAP (0.81, 0.67), OPN (0.24, 0.34), HGF (0.83, 0.62), CCL2 (0.61, 0.24), and OSM (NA, NA).

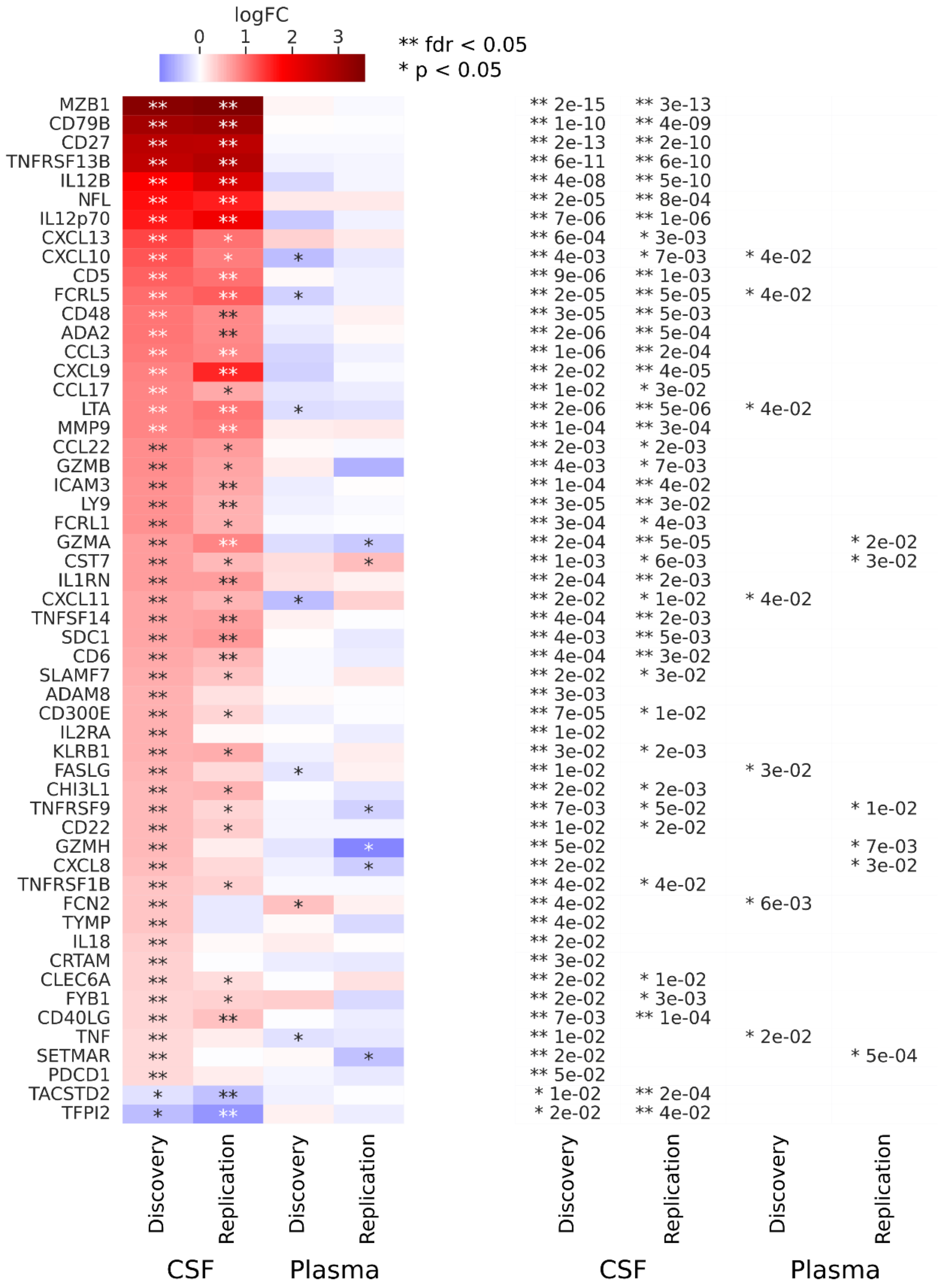

Figure S 5. Heatmap of differentially expressed proteins in cerebrospinal fluid (CSF) and plasma in persons with MS vs. healthy controls.

A heatmap showing the log_2 fold-change (FC) values of the differentially expressed proteins (false discovery rate (FDR) < 0.05) either in the discovery cohort or the replication cohort in the CSF, comparing persons with MS to healthy controls. The log_2FC values in the CSF are compared to the log_2FC values in plasma. The differential expression analysis was performed using a two-sided linear model t-test (Limma analysis).

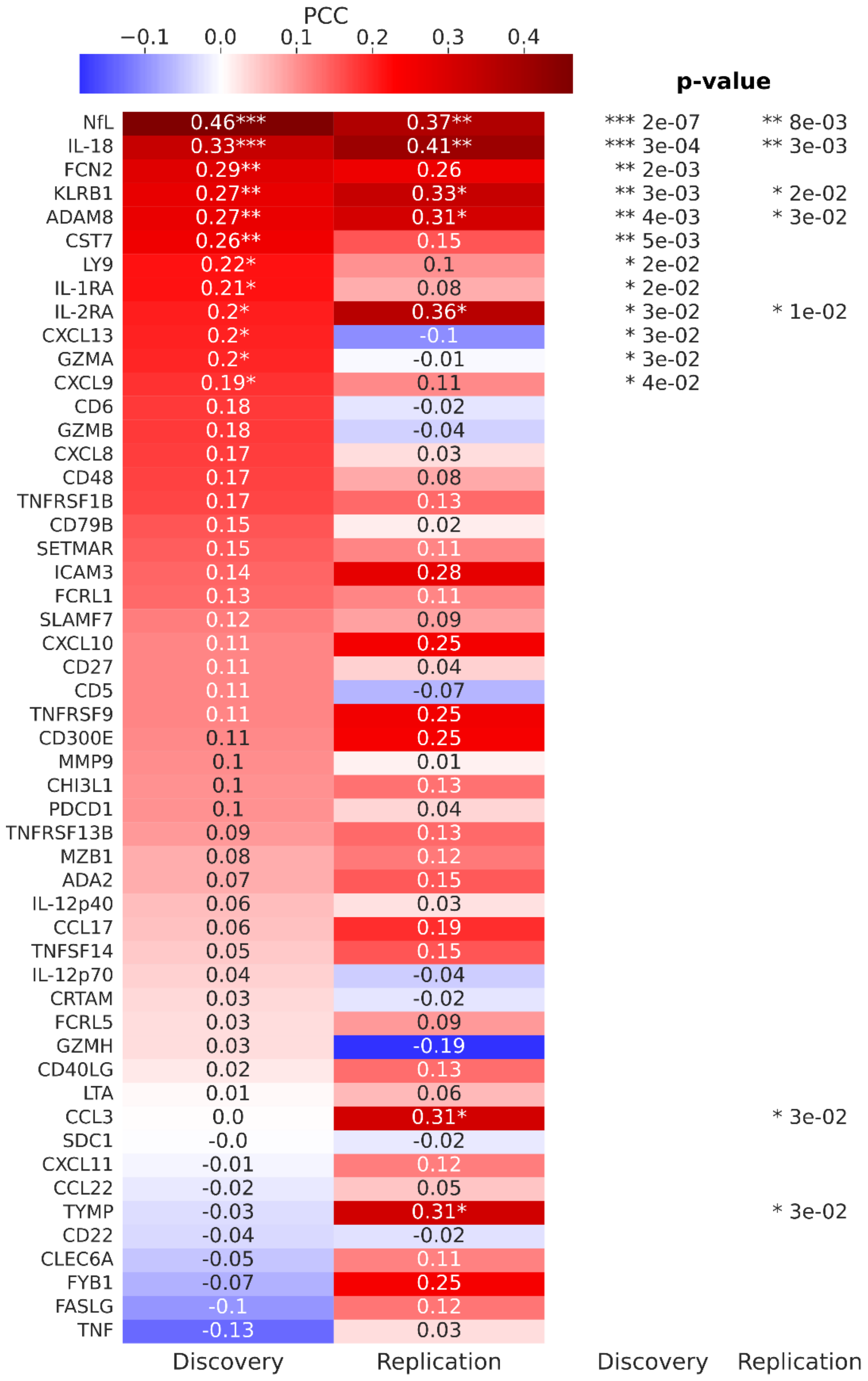

Figure S 6. The correlation between cerebrospinal fluid samples and plasma samples. The correlation, assessed with Pearson's correlation coefficient (PCC), for each of the 52 differentially expressed proteins in the discovery cohort are shown. The significance of the PCCs was assessed with t-statistics (two-sided): * p < 0.05, ** p < 0.01, *** p < 0.001.

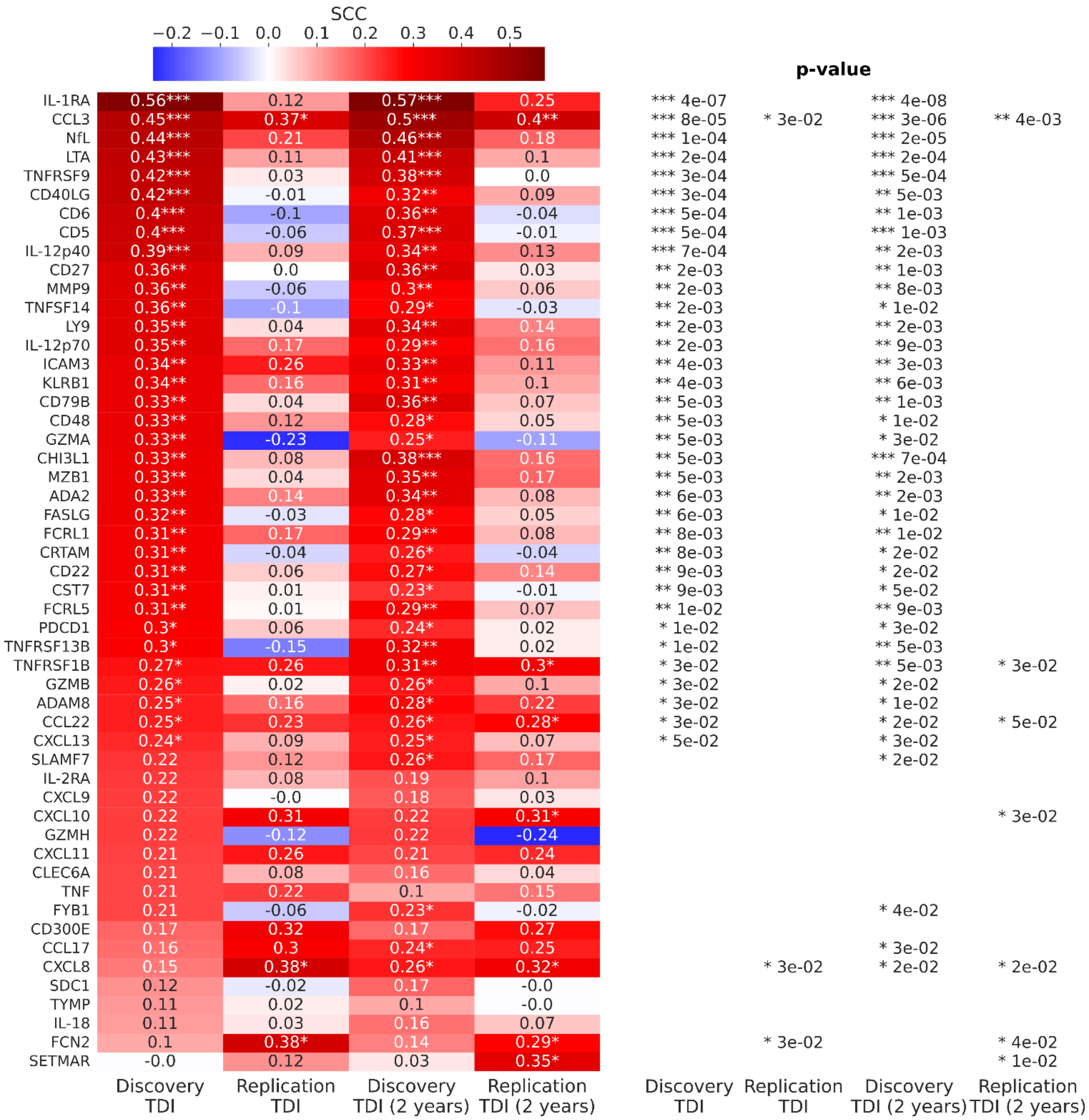

Figure S 7. Correlations between treatment duration index and protein expression. The correlations, assessed with Spearman's correlation coefficient (SCC), between treatment duration index and the expression of each of the 52 differentially expressed proteins in the discovery cohort. For the normalized age-related MS score (nARMSS) models the treatment duration index is based on the full follow-up time from baseline and for the disease activity models the treatment duration index is based on two years from baseline. The significance of the SCCs was assessed with t-statistics (two-sided): * $p < 0.05$, ** $p < 0.01$, *** $p < 0.001$.

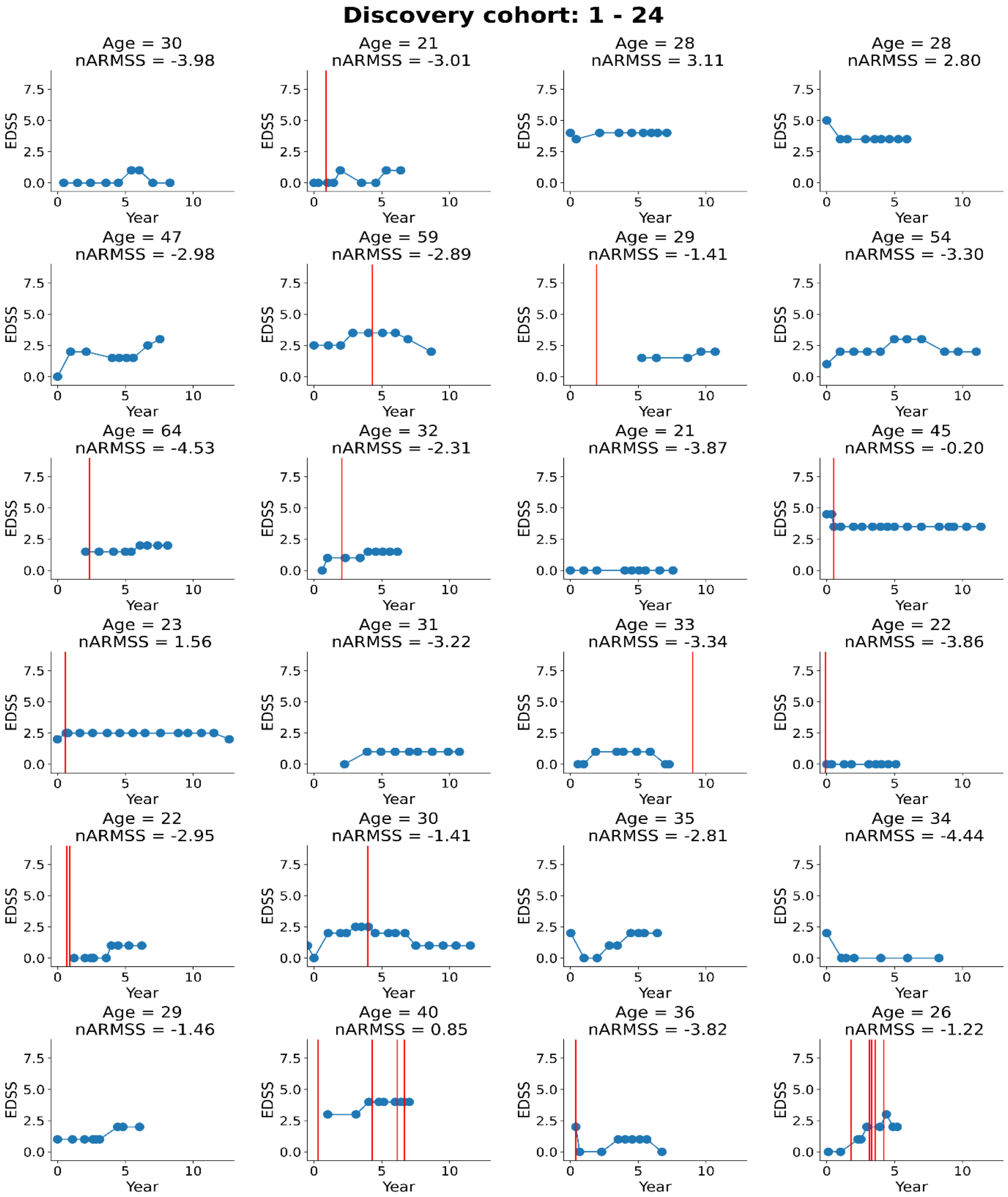

Figure S 8. Individual plots: expanded disability status scale (EDSS) scores of persons with MS.

Individual plots for each person with MS showing the person's EDSS scores (blue dots) during the follow-up years after baseline sampling (discovery cohort: n = 71 samples; replication cohort: n = 33 samples). Plots are shown for persons with at least three years follow-up. Documented relapses are shown with a red vertical line. On top of each plot the person's age at baseline sampling and the calculated normalized age-related MS scores (nARMSS) are stated.

Figure S 8.

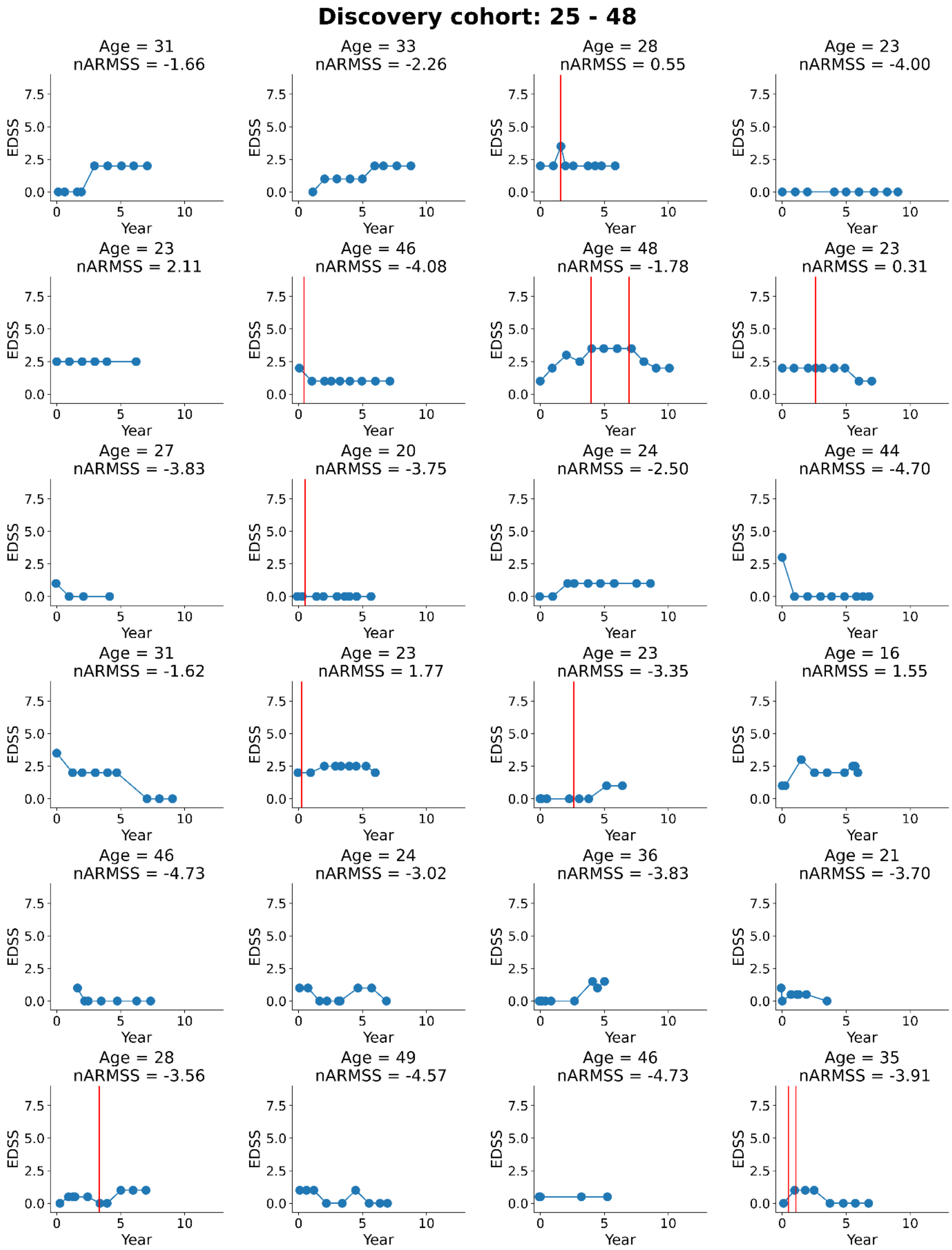
Discovery cohort: 25 - 48
Age = 31
nARMSS = -1.66
Age = 33
nARMSS = -2.26
Age = 28
nARMSS = 0.55
Age = 23
nARMSS = -4.00
Age = 23
nARMSS = 2.11
Age = 46
nARMSS = -4.08
Age = 48
nARMSS = -1.78
Age = 23
nARMSS = 0.31
Age = 27
nARMSS = -3.83
Age = 20
nARMSS = -3.75
Age = 24
nARMSS = -2.50
Age = 44
nARMSS = -4.70
Age = 31
nARMSS = -1.62
Age = 23
nARMSS = 1.77
Age = 23
nARMSS = -3.35
Age = 16
nARMSS = 1.55
Age = 46
nARMSS = -4.73
Age = 24
nARMSS = -3.02
Age = 36
nARMSS = -3.83
Age = 21
nARMSS = -3.70
Age = 28
nARMSS = -3.56
Age = 49
nARMSS = -4.57
Age = 46
nARMSS = -4.73
Age = 35
nARMSS = -3.91
EDSS
Year
0.0
2.5
5.0
7.5
0
5
10

Figure S 8.

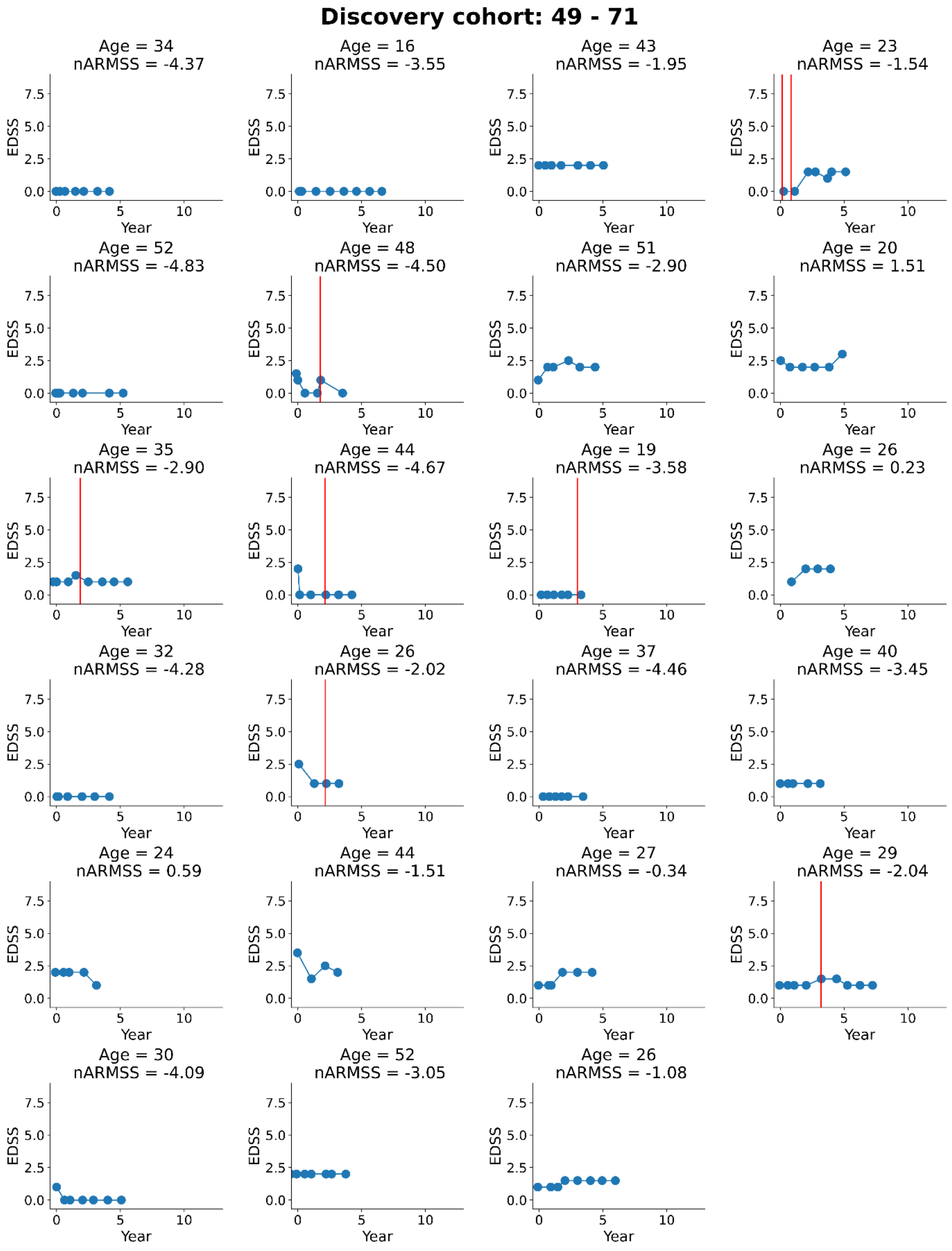
Discovery cohort: 49 - 71
Age = 34
nARMSS = -4.37
Age = 16
nARMSS = -3.55
Age = 43
nARMSS = -1.95
Age = 23
nARMSS = -1.54
Age = 52
nARMSS = -4.83
Age = 48
nARMSS = -4.50
Age = 51
nARMSS = -2.90
Age = 20
nARMSS = 1.51
Age = 35
nARMSS = -2.90
Age = 44
nARMSS = -4.67
Age = 19
nARMSS = -3.58
Age = 26
nARMSS = 0.23
Age = 32
nARMSS = -4.28
Age = 26
nARMSS = -2.02
Age = 37
nARMSS = -4.46
Age = 40
nARMSS = -3.45
Age = 24
nARMSS = 0.59
Age = 44
nARMSS = -1.51
Age = 27
nARMSS = -0.34
Age = 29
nARMSS = -2.04
Age = 30
nARMSS = -4.09
Age = 52
nARMSS = -3.05
Age = 26
nARMSS = -1.08
EDSS
Year

Figure S 8.

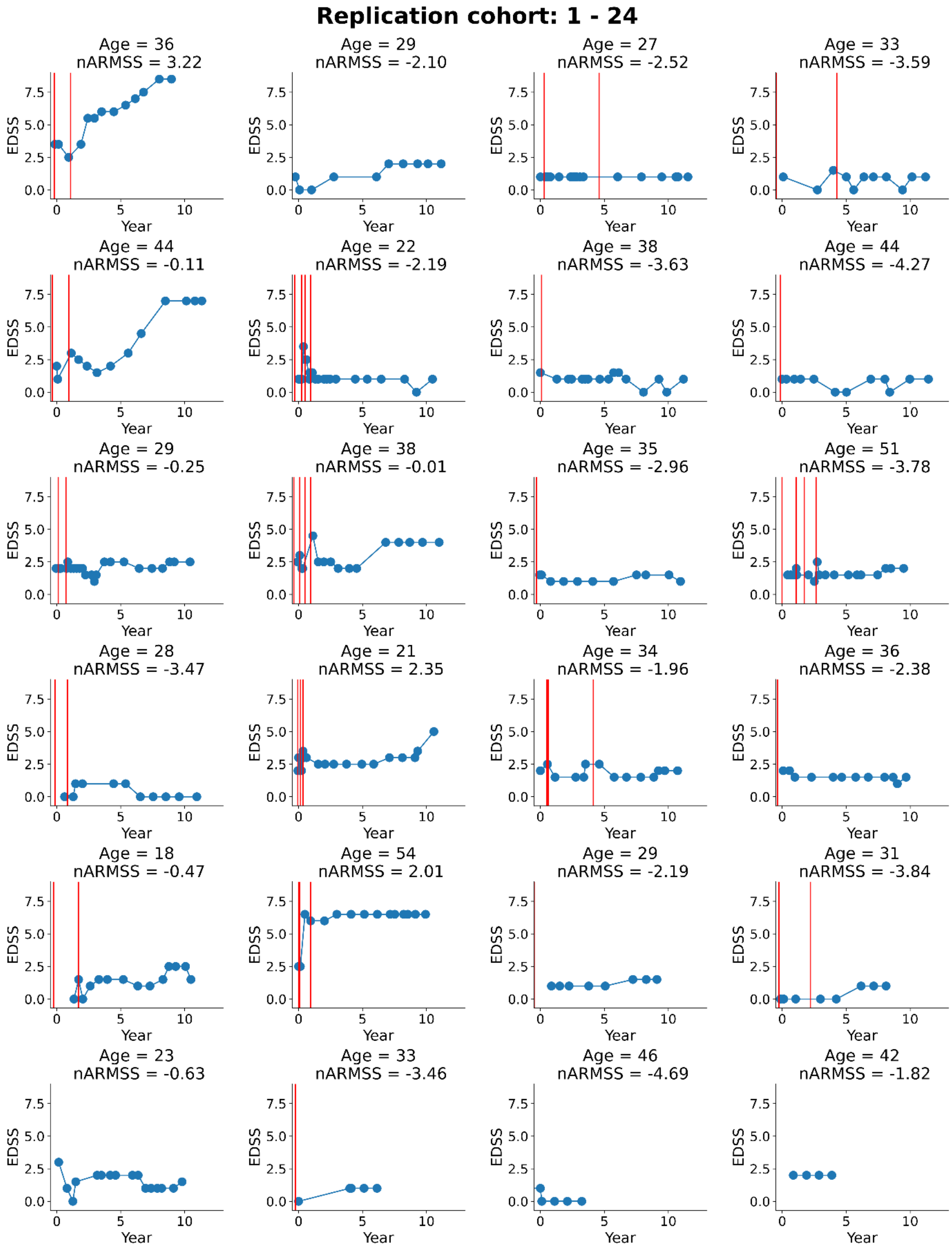

Replication cohort: 1 - 24
Age = 36
nARMSS = 3.22
Age = 29
nARMSS = -2.10
Age = 27
nARMSS = -2.52
Age = 33
nARMSS = -3.59
Age = 44
nARMSS = -0.11
Age = 22
nARMSS = -2.19
Age = 38
nARMSS = -3.63
Age = 44
nARMSS = -4.27
Age = 29
nARMSS = -0.25
Age = 38
nARMSS = -0.01
Age = 35
nARMSS = -2.96
Age = 51
nARMSS = -3.78
Age = 28
nARMSS = -3.47
Age = 21
nARMSS = 2.35
Age = 34
nARMSS = -1.96
Age = 36
nARMSS = -2.38
Age = 18
nARMSS = -0.47
Age = 54
nARMSS = 2.01
Age = 29
nARMSS = -2.19
Age = 31
nARMSS = -3.84
Age = 23
nARMSS = -0.63
Age = 33
nARMSS = -3.46
Age = 46
nARMSS = -4.69
Age = 42
nARMSS = -1.82
EDSS
Year

Figure S 8.

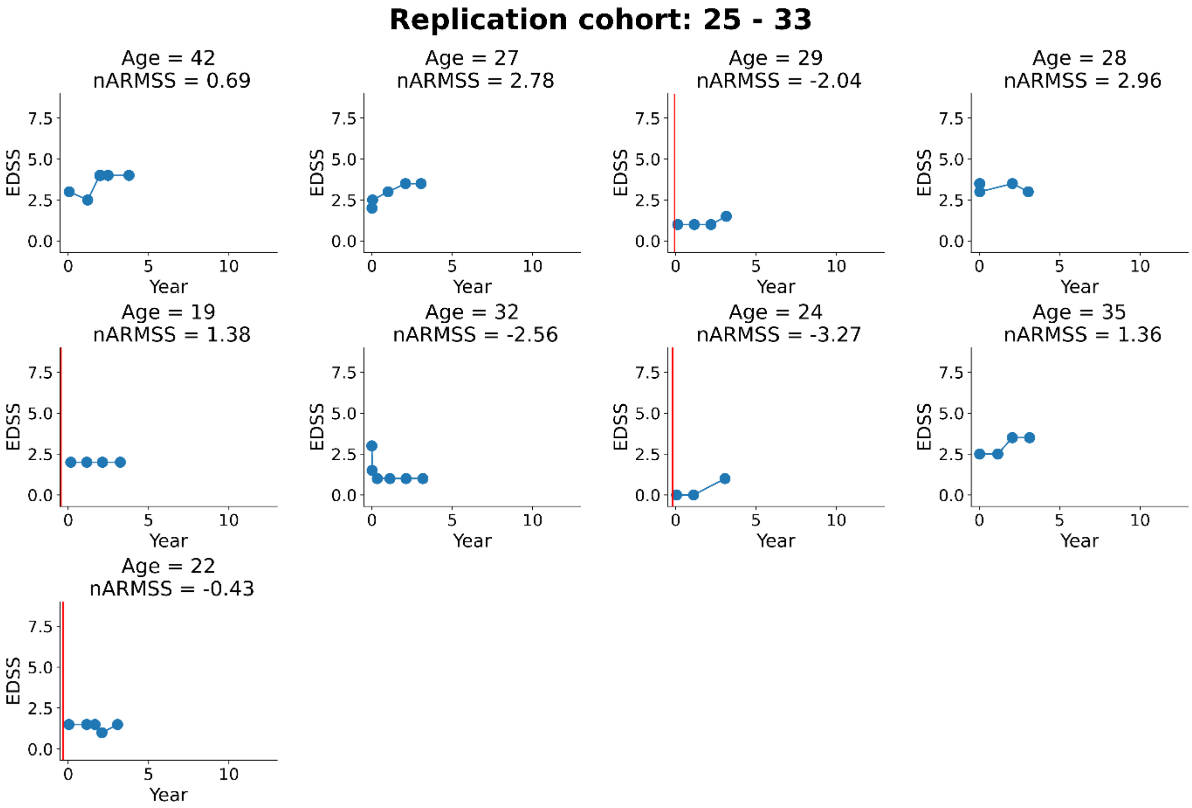
Replication cohort: 25 - 33
Age = 42
nARMSS = 0.69
Age = 27
nARMSS = 2.78
Age = 29
nARMSS = -2.04
Age = 28
nARMSS = 2.96
Age = 19
nARMSS = 1.38
Age = 32
nARMSS = -2.56
Age = 24
nARMSS = -3.27
Age = 35
nARMSS = 1.36
Age = 22
nARMSS = -0.43
EDSS
Year

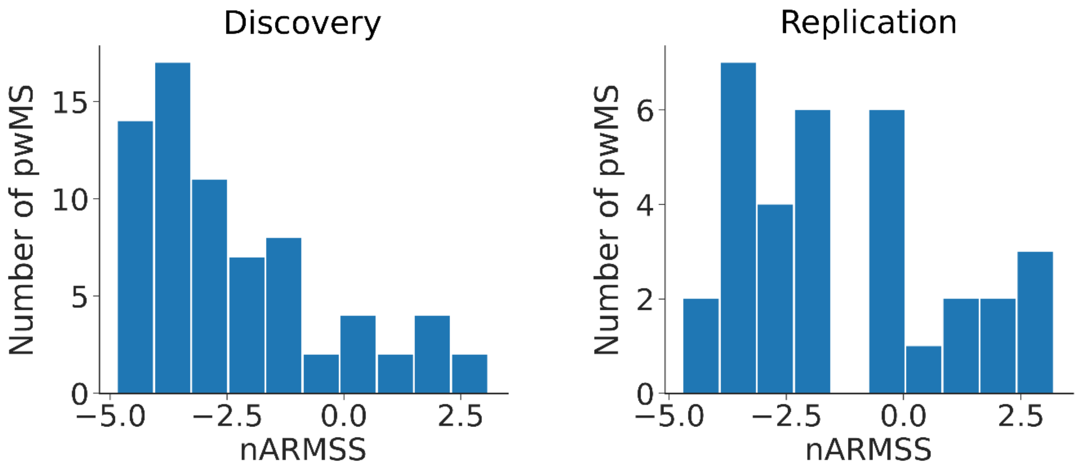

Figure S 9. Normalized age-related MS score (nARMSS) distribution for persons with MS (pwMS).

Histograms showing the distribution of normalized age-related MS scores (nARMSS) for pwMS in the discovery cohort (n = 71 samples; left) and the replication cohort (n = 33 samples; right).

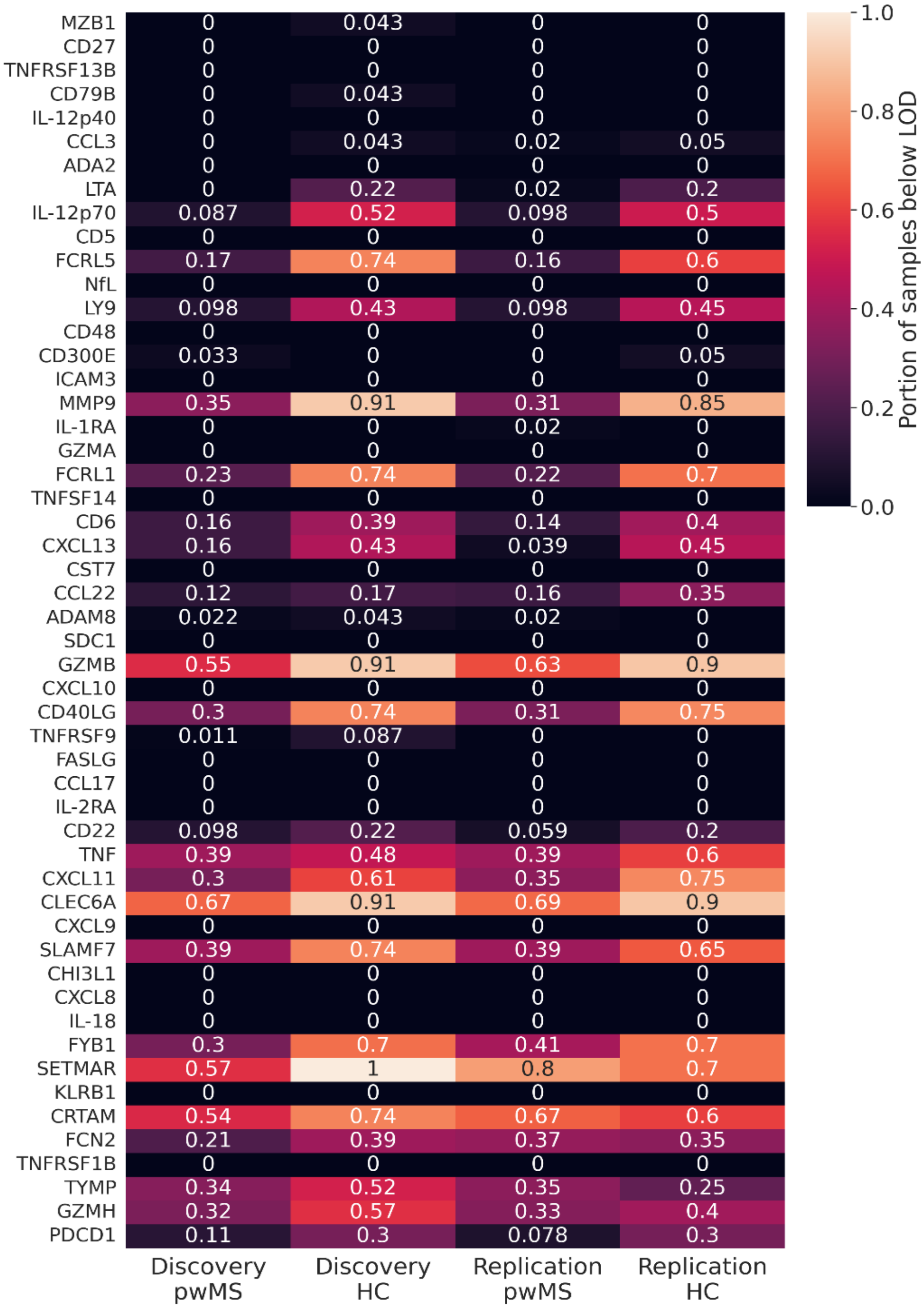

Figure S 10. Portions of samples below the limit of detection (LOD) in persons with MS (pwMS) and healthy controls (HC).

The portions of samples below the LOD in the different groups, pwMS and HC, in the discovery cohort and the replication cohort, separately. The 52 differentially expressed proteins in the discovery cohort are shown.

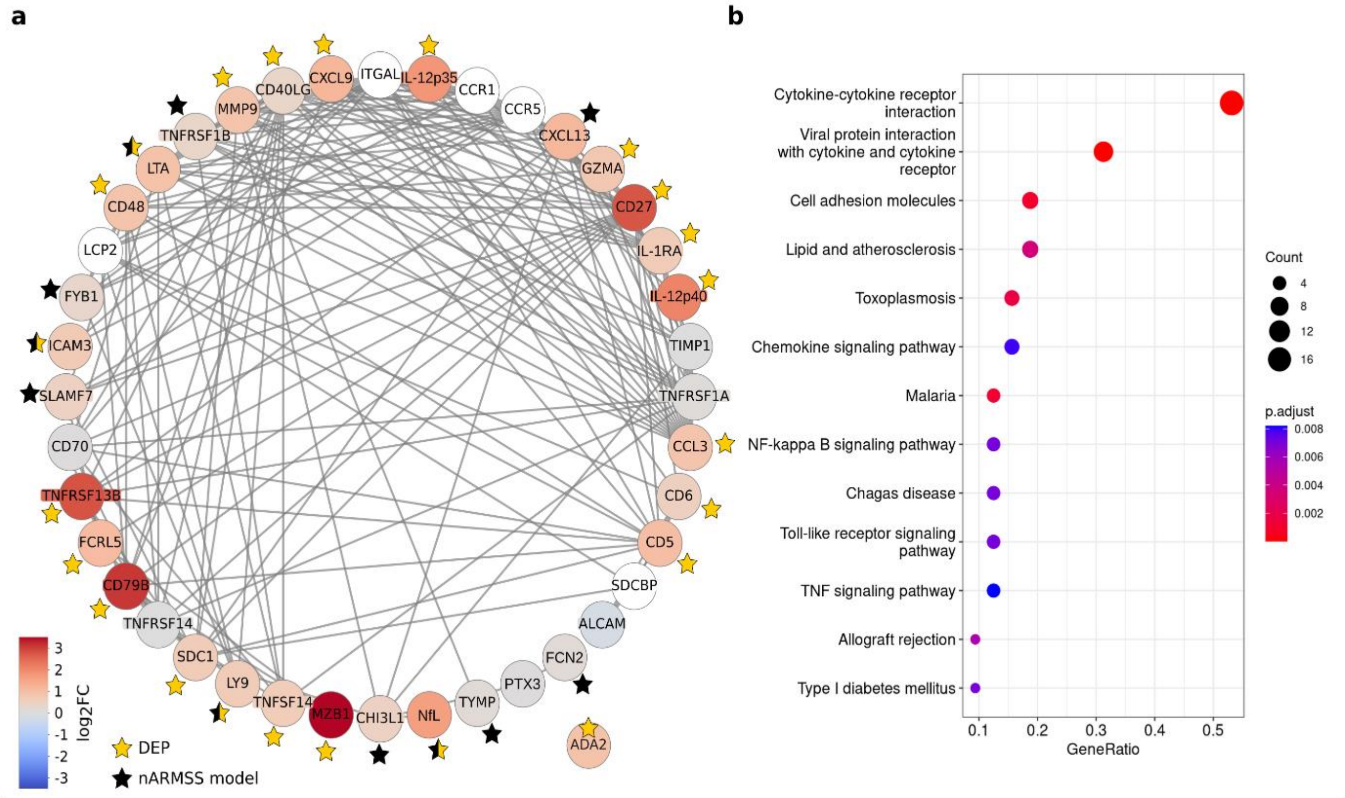

Figure S 11. MS network: circular layout, and KEGG pathway enrichment

a MS network with circular layout. An MS network was formed by connecting the proteins in the normalized age-related MS score (nARMSS) model (black star) and the differentially expressed proteins (DEPs) that overlapped in the discovery and the replication cohort (yellow star). The proteins were connected using STRINGdb (combined interaction score > 0.4) with one intermediate protein allowed to be added to connect proteins. The proteins are color-coded on the $\log_2$ fold change (FC), comparing persons with MS with healthy controls. The white colored proteins were not included in the proteomics profiling. IL-12p35 was not measured in the proteomics profiling but is included as a DEP as it together with IL-12p40 represents IL-12p70. **b** KEGG pathway enrichment of the proteins in the MS network, performed using an over-representation test which is based on a one-sided Fisher's exact test. p-values were corrected for multiple testing (Benjamin-Hochberg). Significant pathways (false discovery rate < 0.05) are shown: cytokine-cytokine receptor interaction (overlap = 17, p = $2*10^{-14}$), viral protein interaction with cytokine and cytokine receptor (overlap = 10, p = $1*10^{-10}$), cell adhesion molecules (overlap = 6, p = $9*10^{-4}$), lipid and atherosclerosis (overlap = 6, p = $4*10^{-3}$), toxoplasmosis (overlap = 5, p = $2*10^{-3}$), chemokine signaling pathway (overlap = 5, p = $8*10^{-3}$), malaria (overlap = 4, p = $1*10^{-3}$), NF-kappa B signaling pathway (overlap = 4, p = $7*10^{-3}$), chagas disease (overlap = 4, p = $7*10^{-3}$), toll-like receptor signaling pathway (overlap = 4, p = $7*10^{-3}$), TNF signaling pathway (overlap = 4, p = $8*10^{-3}$), allograft rejection (overlap = 3, p = $5*10^{-3}$), and type I diabetes mellitus (overlap = 3, p = $7*10^{-3}$).

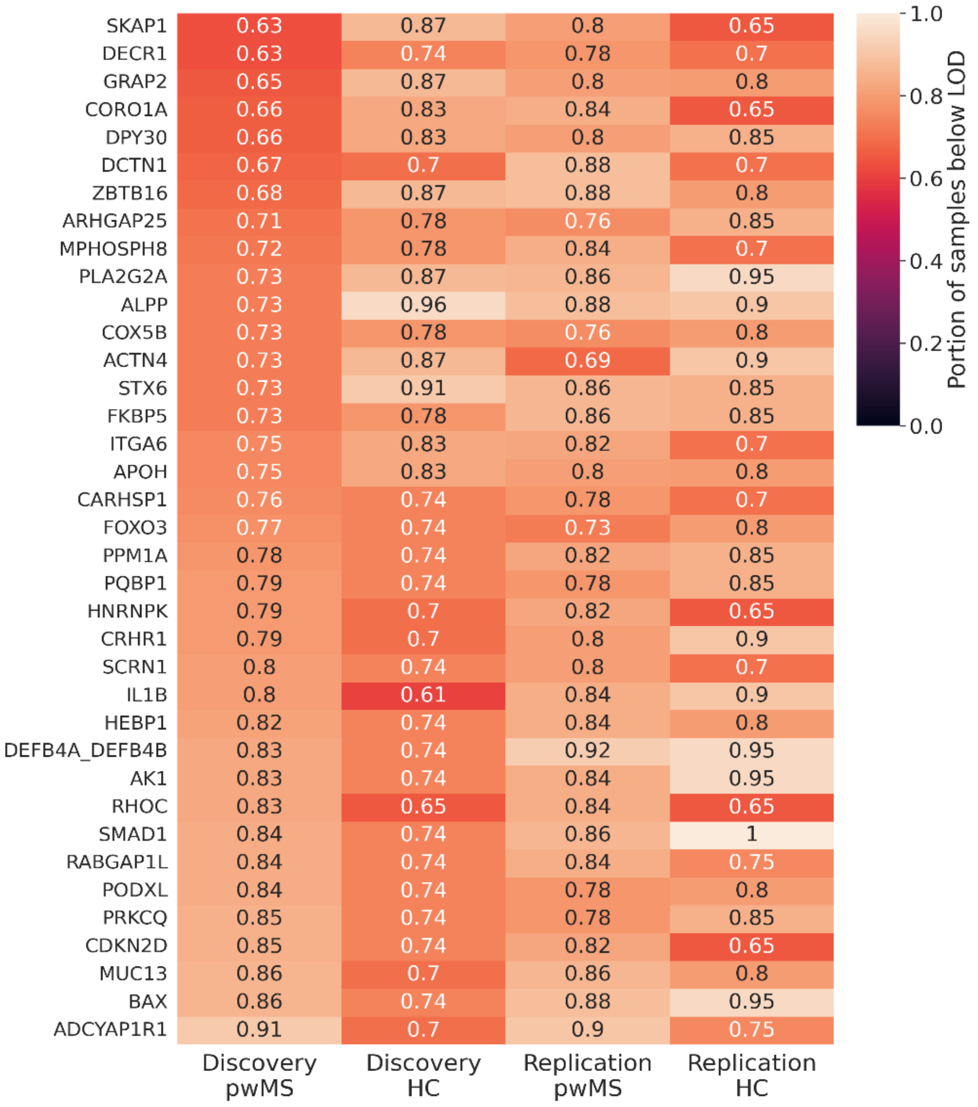

Figure S 12. Portion of samples below limit of detection (LOD) in persons with MS (pwMS) and healthy controls (HC): comparison between discovery and replication cohorts.

The portion of samples below the LOD in the different groups, pwMS and HC, in the discovery cohort and the replication cohort, separately. The figure shows the proteins removed during the pre-processing of the protein data, based on more then 75% of the samples being below LOD, but which had less than 75% of the NPX values below the LOD in either samples from pwMS or HC in the discovery cohort.

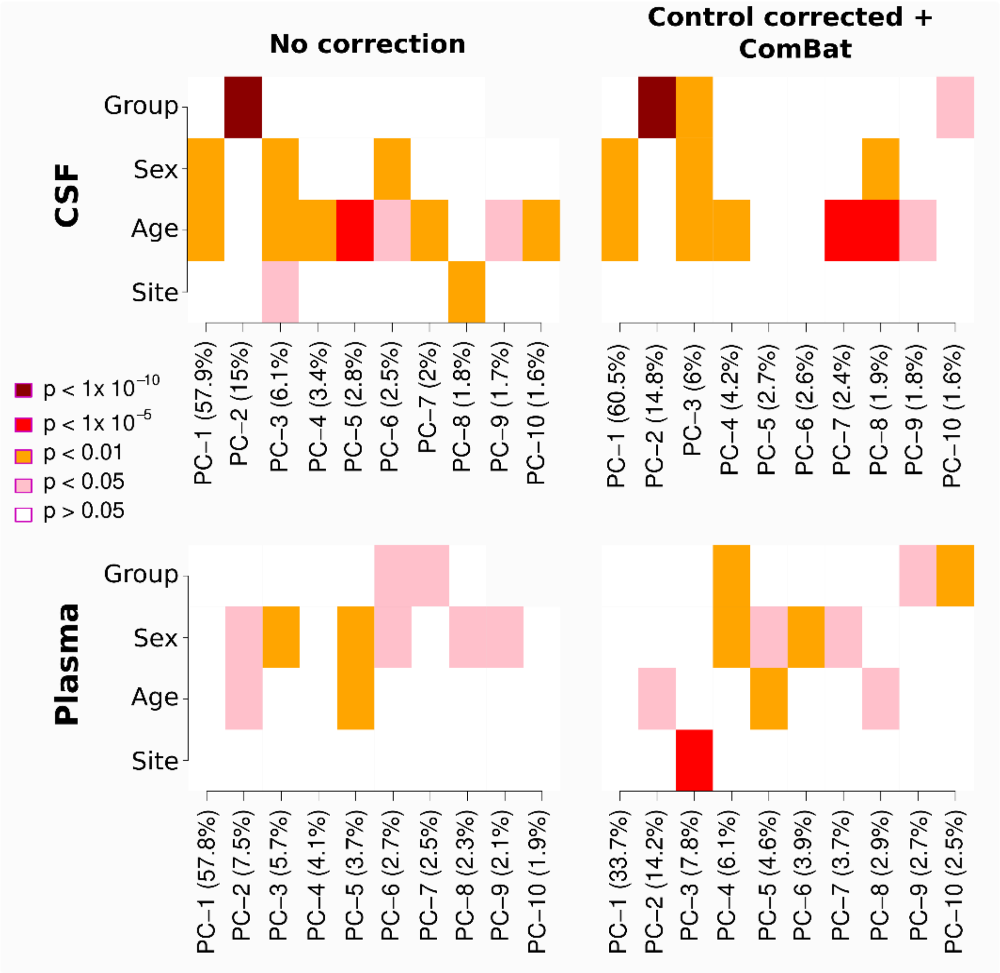

Figure S 13. Singular value decomposition analysis: uncorrected and corrected NPX values in cerebrospinal fluid (CSF) and plasma samples.

Singular value decomposition analysis of the uncorrected NPX values (left) and the corrected NPX values (right) in both the CSF (top) and plasma (bottom) samples. The NPX values were corrected in two steps: 1) control corrected: NPX values were adjusted so that controls in the discovery cohort and the replication cohort had the same standard deviation and mean, 2) ComBat corrected. The categories investigated were group (person with MS or healthy control), sex (female or male), age at baseline, and site (sampled at Linköping university hospital or Karolinska university hospital). The significance of the correlation of each category with each principal component (PC) is shown in the plot.

Table S 1. Known MS biomarkers with supporting references.

Biomarker	Tissue	References
NfL	CSF, plasma	[2-8]
IL-12p40	CSF	[8-10]
C1QA	CSF	[11, 12]
CD27	CSF	[13-15]
CHI3L1	CSF, plasma	[6, 8, 16-18]
CCL2	CSF	[18, 19]
CCL22	CSF	[6, 20]
CXCL1	CSF	[6, 20]
CXCL10	CSF, plasma	[6, 20, 21]
CXCL13	CSF	[6, 7, 18, 22, 23]
MMP9	CSF, plasma	[6, 24]
SPP1 (OPN)	CSF	[9]
CXCL8	CSF	[6]
GFAP	CSF, plasma	[25-32]
CXCL9	CSF	[9]
CCL3	CSF	[9, 33]
OSM	plasma	[9]
HGF	plasma	[9]
CXCL11	CSF	[9]

Table S 2. Performance of the 52 differentially expressed proteins in cerebrospinal fluid in the discovery cohort for predicting MS diagnosis. Area under the curve (AUC) scores in discovery and replication cohorts are shown. The significance of the AUC scores were assessed with a two-sided Mann-Whitney U test.

Protein	Discovery AUC	p-value	Replication AUC	p-value
MZB1	0.985	3.74E-13	0.878	4.16E-07
CD79B	0.974	1.20E-12	0.815	2.09E-05
CD27	0.966	2.64E-12	0.866	9.60E-07
TNFRSF13B	0.958	6.06E-12	0.897	1.17E-07
IL-12p40	0.935	6.13E-11	0.925	1.61E-08
CCL3	0.919	2.98E-10	0.761	3.45E-04
ADA2	0.910	6.57E-10	0.749	5.97E-04
MMP9	0.909	7.17E-10	0.738	9.68E-04
FCRL5	0.898	2.00E-09	0.846	3.30E-06
LTA	0.895	2.58E-09	0.821	1.50E-05
CD5	0.889	4.26E-09	0.772	2.04E-04
IL-12p70	0.886	5.92E-09	0.882	3.20E-07
CD48	0.882	7.88E-09	0.704	4.00E-03
IL-1RA	0.870	2.32E-08	0.844	3.73E-06
LY9	0.862	4.34E-08	0.684	8.27E-03
ICAM3	0.860	5.06E-08	0.662	1.77E-02
FCRL1	0.856	6.88E-08	0.607	8.27E-02
CXCL13	0.853	8.64E-08	0.471	6.52E-01
NfL	0.846	1.57E-07	0.794	6.45E-05
GZMA	0.836	3.39E-07	0.717	2.41E-03
CCL22	0.836	3.39E-07	0.588	1.26E-01
CST7	0.836	3.51E-07	0.664	1.67E-02
TNFSF14	0.834	3.91E-07	0.730	1.36E-03
CD300E	0.815	1.58E-06	0.687	7.44E-03
TNFRSF9	0.808	2.62E-06	0.616	6.66E-02
FASLG	0.804	3.52E-06	0.567	1.94E-01
CD40LG	0.802	4.01E-06	0.770	2.25E-04
CCL17	0.802	4.15E-06	0.622	5.72E-02
CD6	0.799	5.04E-06	0.775	1.77E-04
ADAM8	0.793	7.40E-06	0.617	6.49E-02
SDC1	0.787	1.08E-05	0.744	7.45E-04
CXCL10	0.785	1.22E-05	0.646	2.88E-02
FYB1	0.780	1.77E-05	0.676	1.09E-02
PDCD1	0.778	1.94E-05	0.537	3.16E-01
CXCL11	0.772	2.95E-05	0.614	6.99E-02
IL-2RA	0.765	4.45E-05	0.541	2.98E-01
CXCL8	0.749	1.16E-04	0.648	2.72E-02
FCN2	0.745	1.44E-04	0.404	8.96E-01

SETMAR	0.745	1.48E-04	0.385	9.33E-01
CHI3L1	0.743	1.61E-04	0.726	1.61E-03
TNFRSF1B	0.741	1.89E-04	0.632	4.28E-02
CXCL9	0.740	1.94E-04	0.851	2.44E-06
GZMH	0.739	2.04E-04	0.518	4.11E-01
CD22	0.739	2.10E-04	0.693	6.01E-03
GZMB	0.738	2.21E-04	0.726	1.61E-03
IL-18	0.736	2.39E-04	0.544	2.85E-01
CLEC6A	0.733	2.80E-04	0.635	3.94E-02
SLAMF7	0.731	3.26E-04	0.684	8.27E-03
KLRB1	0.715	7.60E-04	0.678	1.02E-02
CRTAM	0.705	1.25E-03	0.541	2.98E-01
TYMP	0.697	1.79E-03	0.437	7.95E-01
TNF	0.681	3.67E-03	0.629	4.64E-02

Table S 3. Performance of the 52 differentially expressed proteins in cerebrospinal fluid in the discovery cohort for predicting disease activity. Area under the curve (AUC) scores in discovery and replication cohorts are shown. The significance of the AUC scores were assessed with a two-sided Mann-Whitney U test.

Protein	Discovery AUC	p-value	Replication AUC	p-value
NfL	0.748	8.83E-05	0.773	2.31E-02
IL-1RA	0.694	1.91E-03	0.778	2.12E-02
FASLG	0.689	2.61E-03	0.653	1.40E-01
CCL3	0.680	3.97E-03	0.769	2.51E-02
CD6	0.680	3.70E-03	0.533	4.13E-01
ADA2	0.675	4.61E-03	0.756	3.20E-02
CXCL11	0.661	8.39E-03	0.604	2.34E-01
CHI3L1	0.651	1.24E-02	0.644	1.55E-01
TNFRSF1B	0.650	1.36E-02	0.747	3.74E-02
CXCL10	0.647	1.46E-02	0.622	1.97E-01
ICAM3	0.645	1.58E-02	0.756	3.20E-02
TNFSF14	0.638	2.05E-02	0.644	1.55E-01
FCRL5	0.637	2.18E-02	0.796	1.48E-02
GZMH	0.635	2.26E-02	0.529	4.25E-01
LTA	0.635	2.32E-02	0.711	6.58E-02
CXCL13	0.631	2.69E-02	0.613	2.15E-01
GZMA	0.628	2.96E-02	0.578	2.97E-01
TNFRSF13B	0.627	3.03E-02	0.649	1.48E-01
CD48	0.622	3.65E-02	0.627	1.88E-01
CD5	0.621	3.73E-02	0.649	1.48E-01
TNF	0.618	4.13E-02	0.649	1.48E-01
KLRB1	0.617	4.18E-02	0.702	7.49E-02
CCL22	0.613	4.76E-02	0.502	5.00E-01
IL-2RA	0.613	4.87E-02	0.689	9.00E-02
ADAM8	0.608	5.57E-02	0.769	2.51E-02
CRTAM	0.606	6.01E-02	0.858	3.26E-03
TNFRSF9	0.605	6.13E-02	0.749	3.63E-02
IL-12p40	0.605	6.13E-02	0.738	4.34E-02
MZB1	0.603	6.39E-02	0.764	2.73E-02
CD79B	0.603	6.39E-02	0.707	7.02E-02
CLEC6A	0.600	7.06E-02	0.729	5.01E-02
FCRL1	0.600	7.06E-02	0.684	9.55E-02
CXCL9	0.599	7.31E-02	0.724	5.29E-02
CD27	0.598	7.49E-02	0.733	4.67E-02
CST7	0.597	7.64E-02	0.556	3.53E-01
GZMB	0.597	7.79E-02	0.813	1.01E-02
CD40LG	0.597	7.79E-02	0.587	2.75E-01
CD22	0.597	7.79E-02	0.578	2.97E-01

FYB1	0.595	8.09E-02	0.742	4.04E-02
CCL17	0.593	8.57E-02	0.673	1.07E-01
FCN2	0.593	8.52E-02	0.636	1.71E-01
IL-12p70	0.592	8.73E-02	0.796	1.48E-02
LY9	0.592	8.73E-02	0.782	1.94E-02
CD300E	0.591	9.06E-02	0.631	1.80E-01
SLAMF7	0.589	9.58E-02	0.693	8.48E-02
MMP9	0.589	9.58E-02	0.662	1.26E-01
SDC1	0.583	1.13E-01	0.764	2.73E-02
CXCL8	0.583	1.13E-01	0.609	2.25E-01
IL-18	0.553	2.17E-01	0.413	7.35E-01
TYMP	0.529	3.36E-01	0.636	1.71E-01
PDCD1	0.524	3.62E-01	0.667	1.20E-01
SETMAR	0.481	6.11E-01	0.418	7.25E-01

Table S 4. A linear regression model consisting of 11 cerebrospinal fluid (CSF) proteins could predict normalized age-related MS score (nARMSS). The coefficients of the combined model are compared to the predictive power of the individual proteins. For the individual protein models, the coefficient and performance of each model is stated. The performance is assessed with Spearman's correlation coefficient (SCC) and Lin's concordance correlation coefficient (CCC) between the true nARMSS and predicted nARMSS. Age was included as a predictor in the models for all individual proteins. Last, the expression in CSF for four out of the 11 proteins were correlating with the expression in plasma, assessed with Pearson's correlation coefficient. All values are marked for significance, assessed with t-statistics (two-sided): * $p < 0.05$, ** $p < 0.01$, *** $p < 0.001$. All significant p-values ($p < 0.05$) for the stepwise model coefficients were: CXCL13 ($3*10^{-4}$), Age ($9*10^{-4}$), LTA ($9*10^{-4}$), FCN2 (0.002), ICAM3 (0.003), LY9 (0.008), SLAMF7 (0.01), TYMP (0.02), CHI3L1 (0.02), FYB1 (0.03), TNFRSF1B (0.04), and NfL (0.04). For the individual protein model coefficients, the significant p-values were: Age ([$6*10^{-4}$, $2*10^{-3}$]), SLAMF7 (0.04), and NfL (0.04). For the performance scores (SCC, CCC) the significant p-values for the discovery cohort were: CXCL13 (0.001, 0.03), age (0.001, 0.04), LTA ($5*10^{-4}$, 0.02), FCN2 (0.001, 0.02), ICAM3 (0.001, 0.03), LY9 ($2*10^{-4}$, 0.01), SLAMF7 ($3*10^{-5}$, 0.006), TYMP ($4*10^{-4}$, 0.03), CHI3L1 (0.002, 0.03), FYB1 (0.001, 0.03), TNFRSF1B (0.001, 0.01), NfL ($9*10^{-5}$, 0.006). Lastly, the significant p-values for the correlations between CSF and plasma samples were: CXCL13 (0.01), FCN2 (0.03), CHI3L1 (0.02), NfL ($7*10^{-5}$).

	**Stepwise model**	**Individual prediction power**					
			**Discovery**		**Replication**		**Correlation**
**Feature**	**Coefficient**	**Coefficient**	**SCC**	**CCC**	**SCC**	**CCC**	**CSF vs Plasma**
CXCL13	-0.490***	-0.026	0.38**	0.25*	0.24	0.08	0.30*
Age	-0.067***	[-0.068, -0.076]**	0.38**	0.25*	0.23	0.08	
LTA	0.741***	0.192	0.40***	0.28*	0.25	0.1	0.21
FCN2	0.576**	0.153	0.39***	0.27*	0.27	0.11	0.25*
ICAM3	-0.693**	0.052	0.38**	0.25*	0.26	0.09	0.04
LY9	0.717**	0.23	0.42***	0.29*	0.3	0.12	0.14
SLAMF7	0.705*	0.428*	0.48***	0.32**	0.25	0.14	0.05
TYMP	-0.614*	-0.14	0.41***	0.26*	0.21	0.08	-0.09
CHI3L1	-0.827*	0.161	0.37**	0.26*	0.21	0.08	0.27*
FYB1	-0.346*	-0.047	0.38**	0.25*	0.22	0.08	-0.16
TNFRSF1B	0.759*	0.325	0.40***	0.29*	0.34	0.12	0.17
NfL	0.454*	0.369*	0.45***	0.32**	0.14	0.06	0.45***
Intercept	-1.067	[-0.707, 0.111]					

Table S 5. The performance of the normalized age-related MS score (nARMSS) model after exclusion of three proteins. We investigated the nARMSS model (CXCL13, LTA, FCN2, ICAM3, LY9, SLAMF7, TYMP, CHI3L1, FYB1, TNFRSF1B, NfL) with combinations of the proteins SLAMF7, TYMP, and FYB1 removed. These proteins had less than 75% of the samples from persons with MS above the limit of detection (LOD) in the discovery cohort (FYB1, 70% > LOD; TYMP, 66% > LOD; SLAMF7, 61% > LOD). The performance is assessed with Spearman's correlation coefficient (SCC) and Lin's concordance correlation coefficient (CCC) between the true nARMSS and predicted nARMSS. All values are marked for significance, assessed with t-statistics (two-sided): * $p < 0.05$, ** $p < 0.01$, *** $p < 0.001$. All significant p-values ($p < 0.05$) for the correlation coefficients (Discovery SCC, Discovery CCC, Replication SCC, Replication CCC) were: no removed proteins ($3*10^{-11}$, $2*10^{-12}$, $7*10^{-7}$, 0.002), (SLAMF7, TYMP, FYB1) ($2*10^{-8}$, $3*10^{-8}$, 0.001, 0.03), (TYMP, FYB1) ($4*10^{-9}$, $2*10^{-9}$, $9*10^{-4}$, 0.02), (SLAMF7, FYB1) ($7*10^{-10}$, 2*10-9, 1*10-4, 0.008), (SLAMF7, TYMP) ($8*10^{-9}$, $2*10^{-9}$, $5*10^{-4}$, 0.01), FYB1 ($1*10^{-10}$, $4*10^{-11}$, $3*10^{-6}$, 0.005), TYMP ($7*10^{-10}$, $7*10^{-11}$, $3*10^{-4}$, 0.008), SLAMF7 ($3*10^{-10}$, $1*10^{-10}$, $2*10^{-5}$, 0.003).

	**Discovery**		**Replication**	
**Removed proteins**	**SCC**	**CCC**	**SCC**	**CCC**
	0.69***	0.72***	0.74***	0.51**
SLAMF7, TYMP, FYB1	0.61***	0.60***	0.54***	0.37*
TYMP, FYB1	0.63***	0.64***	0.55***	0.39*
SLAMF7, FYB1	0.65***	0.63***	0.62***	0.45**
SLAMF7, TYMP	0.62***	0.64***	0.57***	0.43*
FYB1	0.68***	0.68***	0.72***	0.47**
TYMP	0.65***	0.68***	0.59***	0.45**
SLAMF7	0.66***	0.67***	0.67***	0.49**

Table S 6. References for proteins in the MS network that were not annotated with a Gene Ontology term.

Protein	Functional category	Reference
CHI3L1	Apoptotic processes	[34]
LCP2	T-cell and B-cell activation	[35]
TIMP1	Apoptotic processes	[36]
FCRL5	T-cell and B-cell activation	[37]
FYB	T-cell and B-cell activation	[38, 39]

Table S 7. References for the drug classification into first-line (low-efficacy) and second-line (high-efficacy).

First-line treatments	Second-line treatments
interferon beta-1a [40]	Rituximab [41]
Copaxone [40]	Natalizumab [40, 41]
human normal immunoglobulin (IVIg) [42]*	Fingolimod [40, 41]
dimethyl fumarate [40]	Cladribine [40, 41]
Teriflunomide [40]	Siponimod [43]
Solu-Medrol [44]	Daclizumab [40]
Laquinimod [45]*	Hematopoietic stem cell transplantation [40]
	Ofatumumab [46]
	Ocrelizumab [40, 41]
	Mitoxantrone [40]

*These treatments have never become approved for MS treatment and thus were included in the first-line (less effective treatment) group.

A document from the Swedish MS Association that categorizes DMTs into low, medium or high efficacy (only available in Swedish). On pages 3 and 4, the treatment classifications are listed as low- (lägre effekt), medium- (måttlig effekt) or high- (hög effekt) efficacy. We included the low effect drugs as first-line treatment and the high effect drugs as second-line treatment in our study. We classified the medium effect drugs as either first-line or second-line based on the literature.

https://www.mssallskapet.se/wp-content/uploads/2023/04/SMSS-Nationella-riktlinjer-for-vard-vid-multipel-skleros-230412.pdf

References

1. Huang, J., et al., *Assessing the Preanalytical Variability of Plasma and Cerebrospinal Fluid Processing and Its Effects on Inflammation-Related Protein Biomarkers.* Mol Cell Proteomics, 2021. **20**: p. 100157.
2. Disanto, G., et al., *Serum neurofilament light: a biomarker of neuronal damage in multiple sclerosis.* Annals of neurology, 2017. **81**(6): p. 857-870.
3. van den Bosch, A., et al., *Neurofilament Light Chain Levels in Multiple Sclerosis Correlate With Lesions Containing Foamy Macrophages and With Acute Axonal Damage.* Neurology-Neuroimmunology Neuroinflammation, 2022. **9**(3).
4. Harris, S., et al., *Plasma neurofilament light chain concentrations as a biomarker of clinical and radiologic outcomes in relapsing multiple sclerosis: post hoc analysis of phase 3 ozanimod trials.* European Journal of Neurology, 2021. **28**(11): p. 3722-3730.
5. Håkansson, I., et al., *Neurofilament levels, disease activity and brain volume during follow-up in multiple sclerosis.* Journal of neuroinflammation, 2018. **15**(1): p. 1-10.
6. Håkansson, I., et al., *Neurofilament light chain in cerebrospinal fluid and prediction of disease activity in clinically isolated syndrome and relapsing–remitting multiple sclerosis.* European journal of neurology, 2017. **24**(5): p. 703-712.
7. Novakova, L., et al., *NFL and CXCL13 may reveal disease activity in clinically and radiologically stable MS.* Multiple Sclerosis and Related Disorders, 2020. **46**: p. 102463.
8. Masvekar, R., et al., *Cerebrospinal Fluid Biomarkers of Myeloid and Glial Cell Activation Are Correlated With Multiple Sclerosis Lesional Inflammatory Activity.* Front Neurosci, 2021. **15**: p. 649876.
9. Huang, J., et al., *Inflammation-related plasma and CSF biomarkers for multiple sclerosis.* Proceedings of the National Academy of Sciences, 2020. **117**(23): p. 12952-12960.
10. Barbour, C., et al., *Molecular-based diagnosis of multiple sclerosis and its progressive stage.* Annals of neurology, 2017. **82**(5): p. 795-812.
11. Håkansson, I., et al., *Complement activation in cerebrospinal fluid in clinically isolated syndrome and early stages of relapsing remitting multiple sclerosis.* Journal of Neuroimmunology, 2020. **340**: p. 577147.
12. Lindblom, R.P., et al., *Complement Receptor 2 is increased in cerebrospinal fluid of multiple sclerosis patients and regulates C3 function.* Clinical Immunology, 2016. **166**: p. 89-95.
13. Mescheriakova, J.Y., et al., *Soluble CD27 levels in cerebrospinal fluid as a prognostic biomarker in clinically isolated syndrome.* JAMA neurology, 2017. **74**(3): p. 286-292.
14. Magnusson, R., et al., *RNA-sequencing and mass-spectrometry proteomic time-series analysis of T-cell differentiation identified multiple splice variants models that predicted validated protein biomarkers in inflammatory diseases.* Frontiers in molecular biosciences, 2022: p. 892.
15. Ulutekin, C., et al., *B cell depletion attenuates CD27 signaling of T helper cells in multiple sclerosis.* medRxiv, 2022: p. 2022.10. 17.22281079.
16. De Fino, C., et al., *The predictive value of CSF multiple assay in multiple sclerosis: a single center experience.* Multiple Sclerosis and Related Disorders, 2019. **35**: p. 176-181.
17. Hinsinger, G., et al., *Chitinase 3-like proteins as diagnostic and prognostic biomarkers of multiple sclerosis.* Multiple Sclerosis Journal, 2015. **21**(10): p. 1251-1261.

18. Lucchini, M., et al., *CSF CXCL13 and Chitinase 3-like-1 Levels Predict Disease Course in Relapsing Multiple Sclerosis.* Molecular Neurobiology, 2022: p. 1-15.
19. Mahad, D., S. Howell, and M. Woodroofe, *Expression of chemokines in the CSF and correlation with clinical disease activity in patients with multiple sclerosis.* Journal of Neurology, Neurosurgery & Psychiatry, 2002. **72**(4): p. 498-502.
20. Burman, J., et al., *The cerebrospinal fluid cytokine signature of multiple sclerosis: A homogenous response that does not conform to the Th1/Th2/Th17 convention.* Journal of neuroimmunology, 2014. **277**(1-2): p. 153-159.
21. Sørensen, T., et al., *Chemokines CXCL10 and CCL2: differential involvement in intrathecal inflammation in multiple sclerosis.* European journal of neurology, 2001. **8**(6): p. 665-672.
22. Lucchini, M., et al., *CSF CXCL13 and Chitinase 3-like-1 Levels Predict Disease Course in Relapsing Multiple Sclerosis.* Molecular Neurobiology, 2023. **60**(1): p. 36-50.
23. Pachner, A., *The Brave New World of Early Treatment of Multiple Sclerosis: Using the Molecular Biomarkers CXCL13 and Neurofilament Light to Optimize Immunotherapy.* Biomedicines, 2022. **10**(9): p. 2099.
24. Iannetta, M., et al., *Dynamic changes of MMP-9 plasma levels correlate with JCV reactivation and immune activation in natalizumab-treated multiple sclerosis patients.* Scientific reports, 2019. **9**(1): p. 311.
25. Kuhle, J., et al., *Plasma Neurofilament Light Chain and Glial Fibrillary Acidic Protein Levels Are Prognostic of Disability Worsening: A Biosignature That Helps Differentiating Active From Non-active SPMS (2580)*. 2021, AAN Enterprises.
26. Kassubek, R., et al., *GFAP in early multiple sclerosis: A biomarker for inflammation.* Neuroscience letters, 2017. **657**: p. 166-170.
27. Abdelhak, A., et al., *Serum GFAP as a biomarker for disease severity in multiple sclerosis.* Scientific reports, 2018. **8**(1): p. 1-7.
28. Azzolini, F., et al., *Neuroinflammation is associated with GFAP and sTREM2 levels in multiple sclerosis.* Biomolecules, 2022. **12**(2): p. 222.
29. Abdelhak, A., et al., *Glial activation markers in CSF and serum from patients with primary progressive multiple sclerosis: potential of serum GFAP as disease severity marker?* Frontiers in neurology, 2019. **10**: p. 280.
30. Ayrignac, X., et al., *Serum GFAP in multiple sclerosis: correlation with disease type and MRI markers of disease severity.* Scientific Reports, 2020. **10**(1): p. 10923.
31. Barro, C., et al., *Serum GFAP and NfL levels differentiate subsequent progression and disease activity in patients with progressive multiple sclerosis.* Neurology-Neuroimmunology Neuroinflammation, 2023. **10**(1).
32. Axelsson, M., et al., *Glial fibrillary acidic protein: a potential biomarker for progression in multiple sclerosis.* Journal of neurology, 2011. **258**: p. 882-888.
33. Puthenparampil, M., et al., *Wide cytokine analysis in cerebrospinal fluid at diagnosis identified CCL-3 as a possible prognostic factor for multiple sclerosis.* Frontiers in Immunology, 2020. **11**: p. 174.
34. El Ayoubi, N.K. and S.J. Khoury, *Blood biomarkers as outcome measures in inflammatory neurologic diseases.* Neurotherapeutics, 2017. **14**(1): p. 135-147.
35. Iyer, V.S., et al., *Modulating T-cell activation with antisense oligonucleotides targeting lymphocyte cytosolic protein 2.* Journal of Autoimmunity, 2022. **131**: p. 102857.

36. Ries, C., *Cytokine functions of TIMP-1.* Cellular and molecular life sciences, 2014. **71**(4): p. 659-672.
37. Chorazy, M., et al., *Variants of Novel Immunomodulatory Fc Receptor Like 5 Gene Are Associated With Multiple Sclerosis Susceptibility in the Polish Population.* Frontiers in Neurology, 2021. **12**: p. 631134.
38. Griffiths, E.K., et al., *Positive regulation of T cell activation and integrin adhesion by the adapter Fyb/Slap.* Science, 2001. **293**(5538): p. 2260-2263.
39. Gao, Y., et al., *Identification of gene modules associated with survival of diffuse large B-cell lymphoma treated with CHOP-based chemotherapy.* The pharmacogenomics journal, 2020. **20**(5): p. 705-716.
40. Rotstein, D. and X. Montalban, *Reaching an evidence-based prognosis for personalized treatment of multiple sclerosis.* Nature Reviews Neurology, 2019. **15**(5): p. 287-300.
41. Spelman, T., et al., *Treatment escalation vs immediate initiation of highly effective treatment for patients with relapsing-remitting multiple sclerosis: data from 2 different national strategies.* JAMA neurology, 2021. **78**(10): p. 1197-1204.
42. Živković, S., *Intravenous immunoglobulin in the treatment of neurologic disorders.* Acta Neurologica Scandinavica, 2016. **133**(2): p. 84-96.
43. Kandjani, O.J., et al., *S1PR1 modulators in multiple sclerosis: Efficacy, safety, comparison, and chemical structure insights.* European Journal of Medicinal Chemistry, 2023. **250**: p. 115182.
44. McGinley, M.P., C.H. Goldschmidt, and A.D. Rae-Grant, *Diagnosis and treatment of multiple sclerosis: a review.* Jama, 2021. **325**(8): p. 765-779.
45. Comi, G., et al., *CONCERTO: a randomized, placebo-controlled trial of oral laquinimod in relapsing-remitting multiple sclerosis.* Multiple Sclerosis Journal, 2022. **28**(4): p. 608-619.
46. Svenningsson, A., et al., *Safety and efficacy of rituximab versus dimethyl fumarate in patients with relapsing-remitting multiple sclerosis or clinically isolated syndrome in Sweden: a rater-blinded, phase 3, randomised controlled trial.* The Lancet Neurology, 2022. **21**(8): p. 693-703.
