# Supplementary material for: Proteomics reveal biomarkers for diagnosis, disease activity and long-term disability outcomes in multiple sclerosis
Source: Nat Commun. 2023 Oct 30;14:6903. doi: 10.1038/s41467-023-42682-9 (PMC10616092; doi:10.1038/s41467-023-42682-9)
Supplement: Supplementary file 3 — Description of Additional Supplementary Files [file 41467_2023_42682_MOESM3_ESM.pdf]

Description of Additional Supplementary Files

Supplementary Data 1
Description: Results from differential expression analysis (DEA) of proteins in cerebrospinal fluid (CSF) or plasma, comparing persons with multiple sclerosis to healthy controls.

Supplementary Data 2
Description: Detailed clinical information for each person with multiple sclerosis.
