# Supplementary material for: Proteomics reveal biomarkers for diagnosis, disease activity and long-term disability outcomes in multiple sclerosis
Source: Nat Commun. 2023 Oct 30;14:6903. doi: 10.1038/s41467-023-42682-9 (PMC10616092; doi:10.1038/s41467-023-42682-9)
Supplement: Supplementary file 6 — Reporting Summary [file 41467_2023_42682_MOESM6_ESM.pdf]

nature portfolio

Corresponding author(s): Mika Gustafsson

Last updated by author(s): Sep 27, 2023

Reporting Summary

Nature Portfolio wishes to improve the reproducibility of the work that we publish. This form provides structure for consistency and transparency in reporting. For further information on Nature Portfolio policies, see our Editorial Policies and the Editorial Policy Checklist.

Statistics

For all statistical analyses, confirm that the following items are present in the figure legend, table legend, main text, or Methods section.

n/a	Confirmed	
☐	☒	The exact sample size (*n*) for each experimental group/condition, given as a discrete number and unit of measurement
☐	☒	A statement on whether measurements were taken from distinct samples or whether the same sample was measured repeatedly
☐	☒	The statistical test(s) used AND whether they are one- or two-sided<br>*Only common tests should be described solely by name; describe more complex techniques in the Methods section.*
☐	☒	A description of all covariates tested
☐	☒	A description of any assumptions or corrections, such as tests of normality and adjustment for multiple comparisons
☐	☒	A full description of the statistical parameters including central tendency (e.g. means) or other basic estimates (e.g. regression coefficient) AND variation (e.g. standard deviation) or associated estimates of uncertainty (e.g. confidence intervals)
☐	☒	For null hypothesis testing, the test statistic (e.g. *F*, *t*, *r*) with confidence intervals, effect sizes, degrees of freedom and *P* value noted<br>*Give P values as exact values whenever suitable.*
☒	☐	For Bayesian analysis, information on the choice of priors and Markov chain Monte Carlo settings
☐	☒	For hierarchical and complex designs, identification of the appropriate level for tests and full reporting of outcomes
☐	☒	Estimates of effect sizes (e.g. Cohen's *d*, Pearson's *r*), indicating how they were calculated

Our web collection on statistics for biologists contains articles on many of the points above.

Software and code

Policy information about availability of computer code

Data collection	No software was used for collecting the data
Data analysis	Code used for data analysis is openly available in Zenodo under identifier https://doi.org/10.5281/zenodo.8370589.<br>R 4.2.1<br>R-package ChAMP (v2.21.1): function runCombat<br>R-package Limma (v3.52.4)<br>R-package stats (v 3.6.2): functions glm and step<br>R-package verification (v1.42): function roc.area<br>R-package cutpointr (v1.1.2)<br>R-package clusterProfiler (v4.4.4)<br>Python 3.9.12<br>Python package SciPy (v1.9.1): functions fisher_exact, mannwhitneyu, spearmanr, and integrate.trapezoid<br>Python package scikit-learn (v1.1.2): functions LinearRegression, r2_score, and roc_auc_area<br>Python package statsmodels (v0.13.2): function OLS

For manuscripts utilizing custom algorithms or software that are central to the research but not yet described in published literature, software must be made available to editors and reviewers. We strongly encourage code deposition in a community repository (e.g. GitHub). See the Nature Portfolio guidelines for submitting code & software for further information.

Data

Policy information about availability of data

All manuscripts must include a data availability statement. This statement should provide the following information, where applicable:

- Accession codes, unique identifiers, or web links for publicly available datasets
- A description of any restrictions on data availability
- For clinical datasets or third party data, please ensure that the statement adheres to our policy

The proteomics data generated in this study have been deposited in the DiVA (Digitala Vetenskapliga Arkivet) portal under identifier https://doi.org/10.48360/jcps-gw67. The proteomics data are available under restricted access due to data privacy regulations, access can be obtained by contacting mika.gustafsson@liu.se. Publicly available datasets used in this study: MS-associated genes (C0026769) from DisGeNet version 7.0 (https://www.disgenet.org/), MS SNPs from GWAS (https://doi.org:10.1126/science.aav7188), global ARMSS matrix (https://doi.org:10.1177/1352458517690618), and human protein-protein interactions from STRINGdb version 11.5 (https://string-db.org/). The authors declare that all other data supporting the findings of this study are available within the paper and its supplementary information files. Source data are provided with this paper.

Research involving human participants, their data, or biological material

Policy information about studies with human participants or human data. See also policy information about sex, gender (identity/presentation), and sexual orientation and race, ethnicity and racism.

Reporting on sex and gender	Sex of participants was determined based on Swedish official medical records. Sex distributions are reported within the manuscript (Table 1) and detailed information provided in Supplementary Data 2. Sex-based analyses were performed to determine if investigated groups differed in sex distribution, as well as to determine if sex was a significant covariate in the biomarker models. Healthy controls in the discovery cohort are sex-matched against the patients.
Reporting on race, ethnicity, or other socially relevant groupings	No categorization regarding race, ethnicity or other socially relevant groupings were used in this study.
Population characteristics	Characteristics of persons with MS and healthy controls are provided in Table 1 and Table 2 within the manuscript. These characteristics are specified for both cohorts used in this study. Characteristics are: cohort size, sex, age, routine CSF findings, clinical data including diagnosis, disease duration, treatments, MRI data, evidence of disease activity, etc.
Recruitment	Participants were recruited consecutively at the Department of Neurology among patients referred to this department because of symptoms suggestive of MS and where a clinical investigation for this diagnosis was initiated after evaluation by a neurologist. Patients from both outpatient and inpatient care were included. A possible selection bias could be that most patients were recruited in the outpatient care, that is, the population was dominated by patients having minor to moderate neurologic deficits at inclusion. The cohort characteristics is however described in the manuscript.
Ethics oversight	This study was reviewed and approved by the regional ethics review board in Linköping, Sweden (2013/155-32, 2016/304-32,2016/305-32, 2014/311-31, 2017/288-31) and the ethics review board in Stockholm, Sweden (2022-03650-02). The participants provided their written informed consent to participate in this study.

Note that full information on the approval of the study protocol must also be provided in the manuscript.

Field-specific reporting

Please select the one below that is the best fit for your research. If you are not sure, read the appropriate sections before making your selection.

☒ Life sciences ☐ Behavioural & social sciences ☐ Ecological, evolutionary & environmental sciences

For a reference copy of the document with all sections, see nature.com/documents/nr-reporting-summary-flat.pdf

Life sciences study design

All studies must disclose on these points even when the disclosure is negative.

Sample size	We made every effort to incorporate a maximum number of samples from individuals with MS for whom we had subsequent diagnoses. This was to develop regression models for disease severity across various sites, enabling both replication and discovery analysis. In addition, we included controls according to our previous studies using Olink inflammation panel (Huang, PNAS, 2020). Our cohorts of 143 patients and 43 healthy controls would suffice to detect quite some proteins dysregulated in MS vs HC. Considering the extensive number of samples and analytes examined in the study, coupled with the rigorous statistical models used for multiple cross-comparisons between different cohorts, we are confident that most confounding factors have been effectively addressed.
Data exclusions	Plasma samples from 21 pwMS in the replication cohort had higher expression of several protein markers known to be affected by sampling and handling variability and were therefore excluded from further analysis (see Supplementary Fig. 1).

Replication	The pwMS were divided into a discovery cohort (92 pwMS and 23 HC from Linköping University Hospital) and a replication cohort (51 pwMS and 20 HC from Karolinska University Hospital). All the models in this study were trained in the discovery cohort and tested in the replication cohort. The area under the receiver operating characteristic curve (AUC) and p-value of each model in each cohort and subcohorts are clearly stated in the figures, figure legends and manuscript text.
Randomization	No randomization was performed since this was not an interventional study, but an observational study.
Blinding	The investigators, primarily clinicians who assessed the clinical data and additionally the scientists who analyzed the data, were not blinded to the group allocation in terms of which individuals were patients with MS and which individuals were healthy controls. This information is necessary to know for performing the analyses in every step of the study. All models were trained exclusively on the discovery cohort and subsequently validated on the replication cohort, ensuring no cross-talk or re-adjustment based on the replication cohort data. This strategy facilitated an effective form of blinding at the model level.

Reporting for specific materials, systems and methods

We require information from authors about some types of materials, experimental systems and methods used in many studies. Here, indicate whether each material, system or method listed is relevant to your study. If you are not sure if a list item applies to your research, read the appropriate section before selecting a response.

Materials & experimental systems

n/a	Involved in the study	
☒	☐	Antibodies
☒	☐	Eukaryotic cell lines
☒	☐	Palaeontology and archaeology
☒	☐	Animals and other organisms
☒	☐	Clinical data
☒	☐	Dual use research of concern
☒	☐	Plants

Methods

n/a	Involved in the study	
☒	☐	ChIP-seq
☒	☐	Flow cytometry
☒	☐	MRI-based neuroimaging
